# Aerial Warfare: A Volatile Dialogue between the Plant Pathogen *Verticillium longisporum* and Its Antagonist *Paenibacillus polymyxa*

**DOI:** 10.3389/fpls.2017.01294

**Published:** 2017-07-27

**Authors:** Daria Rybakova, Ute Rack-Wetzlinger, Tomislav Cernava, Angelika Schaefer, Maria Schmuck, Gabriele Berg

**Affiliations:** ^1^Institute of Environmental Biotechnology, Graz University of Technology Graz, Austria; ^2^Austrian Centre of Industrial Biotechnology—GmbH Graz, Austria

**Keywords:** biocontrol, Verticillium wilt, *Verticillium longisporum*, *Paenibacillus polymyxa*, volatile assay, VOCs, plant-microbe interactions, adaptation

## Abstract

Verticillium wilt caused by *Verticillium* spp. results in severe yield losses in a broad range of crops. *Verticillium* outbreaks are challenging to control, and exacerbated by increases in soil temperatures and drought associated with global warming. Employing natural antagonists as biocontrol agents offers a promising approach to addressing this challenge. *Paenibacillus polymyxa* Sb3-1 was proven to reduce the growth of *Verticillium longisporum* during *in vitro* experiments and was shown to promote the growth of oilseed rape seedlings infested with *V. longisporum*. Our novel approach combined *in vitro* and *in planta* methods with the study of the mode of interaction between Sb3-1 and *V. longisporum* EVL43 via their volatile organic compounds (VOCs). Volatile and soluble substances, produced by both microorganisms as a reaction to one another's VOCs, were detected by using both gas and liquid chromatography-mass spectrometry. *P. polymyxa* Sb3-1 continually produced antimicrobial and plant growth promoting VOCs, such as 2-nonanone and 3-hydroxy-2-butanone. Several other antimicrobial volatile substances, such as isoamyl acetate and durenol, were downregulated. The general metabolic activity of Sb3-1, including protein and DNA biotransformations, was upregulated upon contact with EVL43 VOCs. *V. longisporum* increased its production of antimicrobial substances, such as 1-butanol, and downregulated its metabolic activities upon exposure to Sb3-1 VOCs. Additionally, several stress response substances such as arabitol and protein breakdown products (e.g., L-Isoleucyl-L-glutamic acid), were increased in the co-incubated samples. The results obtained depict an ongoing dialog between these microorganisms resulting in growth inhibition, the slowing down of metabolism, and the cell death of *V. longisporum* due to contact with the *P. polymyxa* Sb3-1 VOCs. Moreover, the results indicate that VOCs make a substantial contribution to the interaction between pathogens and their natural antagonists and have the potential to control pathogens in a novel, environmentally friendly manner.

## Introduction

Verticillium wilt caused by *Verticillium* spp. is a serious fungal plant disease that affects up to 250 hosts, including many economically important crops such as potato, strawberry, alfalfa, oilseed crops, and a variety of tree species (Hiemstra, 1998; Pegg and Brady, 2002). While *V. dahliae* Kleb. (1913) and *V. albo-atrum* Reinke and Berthold (1879) attack a high number of host species, *V. longisporum* (C. Stark) Karapapa et al. (1997) has a more defined host range, primarily infesting cruciferous species (*Brassicaceae*). *V. longisporum* is the main contributor to Verticillium wilt in oilseed crops (Zeise and von Tiedemann, 2002; Depotter et al., 2016), and causes up to 50% of yield losses (Daebeler et al., 1988; Zeise and Steinbach, 2004). *Verticillium* spp. are the most challenging types of phytopathogens to control due to the extensive viability of their microsclerotia (resting structures), their broad host range, and the genetically heterogeneous and polyphyletic character of *Verticillium* isolates, as well as the absence of effective fungicide treatments (Fradin et al., 2009; Jiménez-Gasco et al., 2014). Due to the decrease in crop rotation time and global warming (Houghton et al., 2001), the number of disease incidents is expected to increase in the future (Heale and Karapapa, 1999; Siebold and von Tiedemann, 2012). *Verticillium* has an optimum temperature of 20°C and above for most of its life cycle stages (Siebold and von Tiedemann, 2012). In this context, global warming will increase the temperature of the soil's upper layers in which the fungal inoculum is found to be present (Zhang et al., 2005), causing the shifting of soil temperatures toward the biological optimum temperature for the fungus at locations where the disease is currently not a problem. Such temperature-related shifts, or 'fingerprints' in species distribution due to global warming have been observed within a large number of species (Root et al., 2003). In addition, the infection period can be extended at higher temperatures (Dunker et al., 2008). This concurs with recent observations that the wilt disease of the oilseed rape (OSR) has been occurring more frequently in recent, warmer years (Siebold and von Tiedemann, 2012). The Verticillium wilt of strawberry crops is also affected by temperature increases, as was shown by an increase of damage occurring during warm conditions (Schubert et al., 2009). Considering the current trend in plant disease control toward sustainable agriculture and the urgent need for agricultural adaptation to climate change (Howden et al., 2007), environmentally friendly solutions for Verticillium wilt problems, such as biological control, are especially desirable.

Several biocontrol agents (BCAs) such as *Serratia plymuthica* HRO-C48 (Müller and Berg, 2008), non-pathogenic *Verticillium* strains (França et al., 2013), as well as several strains of *Paenibacillus* and *Serratia* (Rybakova et al., 2016b) were proposed as potentially effective biocontrol agents against *V. longisporum*. Endophytic *Paenibacillus* strains are known for their plant growth promoting and biocontrol properties (Rybakova et al., 2016a). *P. polymyxa* Sb3-1 was shown to be particularly active against *V. longisporum in vitro*. This strain demonstrated a plant growth promoting effect on the oilseed rape seedlings in sterile soil, and was deleterious to the seedlings under sterile soil-free conditions (Rybakova et al., 2016b). Several modes of actions are suggested for *Paenibacillus* spp. as biocontrol agents. They are able to protect plants against pathogenic organisms in a variety of ways including the production of a variety of antimicrobials and insecticides and triggering a plant's defense system known as induced systemic resistance (Grady et al., 2016). *Paenibacillus* spp. can also produce a biofilm around plant roots that functions as a protective layer preventing pathogens from accessing plant tissue (Timmusk et al., 2005). Recently, the emission of antimicrobial volatile organic substances (VOCs) has been proposed as an important defense mechanism of *Paenibacillus* (Rybakova et al., 2016a). A multitude of interactions between organisms is based on the emission and perception of volatiles (Wenke et al., 2010). In fact, recent data suggests that the majority of all explored interactions is based on volatile compounds rather than on non-volatile ones (Kanchiswamy et al., 2015). Even plants can sense VOCs produced by neighboring plants (Baldwin et al., 2006; Dicke et al., 2009). Volatiles act as an important medium for interactions between bacteria and fungi below ground (Insam and Seewald, 2010; Effmert et al., 2012). In this context, it was shown that fungi emit a broad spectrum of VOCs with diverse ecological functions affecting both bacteria (Schmidt et al., 2015; Hacquard, 2017) and plants (Ditengou et al., 2015). Bacteria are known to produce highly diverse volatiles (Schulz and Dickschat, 2007). Such microbial VOCs can directly or indirectly mediate increases in the biomass of the host plant. They also increase disease resistance and abiotic stress tolerance, and thereby help plants to combat pathogens (Liu and Zhang, 2015). In terms of microbial interplay it is hypothesized that VOCs can be used either as infochemical molecules affecting the gene expression in the responding microorganism, or as competitive tools, providing an advantage by suppressing or eliminating potential enemies (Kai et al., 2007; Schmidt et al., 2015; Ossowicki et al., 2017). The diversity of soil bacteria and the presence of some less abundant soil bacteria, such as *Paenibacillus*, play important roles in the production of antifungal volatiles by soil bacterial communities (Hol et al., 2015; Schulz-Bohm et al., 2015). Diazine derivatives produced by several *Paenibacillus* spp., for example, are able to suppress the growth of bacteria, fungi and yeast (Cernava, 2012). Due to their great potential for application as a pathogen control in industrial environments a patent was filed for commercial utilization of novel bioactive compounds (Aichner et al., 2012). Currently, various tools are available to access bioactivity of microbial volatiles. These tools include pre-screening methods such as split-plate experiments and parallelized VOCs assays (Ryu et al., 2003; Cernava et al., 2015a). Further characterization of the substances involved requires an analytical approach. Highly concentrated volatiles can be captured with an air tight syringe (headspace method) and injected into a GC-MS system. Alternatively, headspace solid-phase microextraction (SPME) can also capture less abundant volatiles, which is beneficial when general VOC emissions of microorganisms are studied (Zhang and Pawliszyn, 1993).

While in recent years the diversity of microbial volatiles has been studied intensively, the ways in which they contribute to the mode of action of biocontrol agents, and their exact role in specific microbe-microbe interactions remain largely unknown. In order to address this knowledge gap, we studied the interaction between plant pathogenic *Verticillium longisporum* EVL43 and the potential biocontol agent *Paenibacillus polymyxa* Sb3-1 via their volatiles *in vitro* and *in planta*. The main objective of the study was to understand the mode of action of the predicted biocontrol agent *P. polymyxa* Sb3-1 against plant pathogen *V. longisporum* via its volatiles. We hypothesized that the exchange of volatiles plays an important role in the interaction between these two microorganisms and is involved in the antagonistic effect of *P. polymyxa* Sb3-1 against Verticillium wilt.

## Materials and methods

### Bacterial strains and growth conditions

The fungal pathogen used was *V. longisporum* (C. Stark) Karapapa et al. (1997) strains ELV25 and EVL43 from the collection TU Graz, Environmental Biotechnology, described in Messner et al. (1996). *V. longisporum* ELV25 was grown either on potato dextrose agar (PDA) or in Czapek Dox liquid culture (Sigma-Aldrich). *P. polymyxa* Sb3-1 (Köberl et al., 2013) as well as its rifampicillin resistant mutant *P. polymyxa* Sb3-1 rif^R^ (this study) were routinely grown on Standard I nutrient agar (NA, SIFIN, Berlin, Germany) at 30°C. When required, rifampicin was added at concentrations of 100 μg ml^−1^. For the direct dual culture assays, Reasoner's 2A agar (R2A) (Roth, Karlsruhe, Germany), water yeast agar (WAY), and PDA were used.

### Evaluation of the plant growth promotion (PGP) and biocontrol effects of *P. polymyxa* Sb3-1 *In planta*

Oilseed rape (*Brassica napus* L. “Traviata H 605886”; KWS Saat Einbeck, Germany) seeds were treated with two concentrations of *P. polymyxa* Sb3-1 (log_10_ 7 and log_10_ 5 CFU ml^−1^) applied to the seeds using the pelleting method according to the protocol described by Müller and Berg (2008). For the PGP studies, the experiment was performed in 5 replicates with 9 seedlings each (3 seeds per pot). The 2-week old seedlings were harvested and their fresh weight was compared to the untreated control. The bacterial abundance on the seeds and on the roots was estimated as described by Rybakova et al. (2016b). For the biocontrol studies, the 1-week old seedlings were inoculated with *V. longisporum* ELV25 using the root dipping method. The experimental setup included 15 pots per treatment with one plant per pot and replicate. The roots of the 1-week old seedlings were artificially injured using a scalpel. The seedlings were inoculated by dipping their roots for 30 min into 200 ml of Czapek-Dox broth with a 1-week old culture of *V. longisporum* ELV25 adjusted to 10^6^ CFU ml^−1^. Control treatments were treated the same way as the inoculated seedlings, however they were immersed in sterile water instead of *V. longisporum* ELV25 blastospore solution. Finally, all plants were transplanted back to the pots. After the appearance of the first disease symptoms, the disease reaction of plants was assessed based on the severity of symptoms as described by Müller and Berg (2008) at weekly intervals for the duration of 7 weeks after inoculation. Data on disease severity was used to calculate area under disease process curve (AUDPC) determined as AUDPC = Σ((Si + Sti + 1)/2)^*^(ti + 1 − ti), where Si is the symptoms severity, and ti is the date of assessment of symptoms severity.

### Volatile metabolite analyses with *P. polymyxa* Sb3-1 and *V. longisporum* ELV43

Antagonism of *P. polymyxa* Sb3-1 and *V. longisporum* ELV43 was tested with the “Two Clamp VOCs Assay” as described in Cernava et al. (2015a). The assay was performed in nine replicates. Three plates with three wells each were used per replicate. The significance of the differences between zones of inhibition of *Verticillium* growth by different bacterial strains (**Table 2**) was calculated using one-way ANOVA and Tukey's HSD tests. For both analyses, the *P* < 0.05 were considered to be significant.

GC-MS headspace SPME experiments were carried out as described by Cernava et al. (2015a; Supplementary Figure 1). Identification of the volatile compounds was performed with the NIST MS Search 2.2 included in the Software-Package of the NIST 2014 database. Further verification was done by calculation of the covats index (CI) followed by comparisons to database entries of NIST Search 2.2 and the entries in the Online Database of NIST (http://webbook.nist.gov/). The raw data of all analyses is provided as Supplementary Data for this publication; file names include the analyzed microorganisms and the duration of the incubation prior to VOC sampling.

### Soluble metabolite analyses of *P. polymyxa* Sb3-1 and *V. longisporum* ELV43

In the course of an LC-MS assay *V. longisporum* ELV43 and *P. polymyxa* Sb3-1 were co-incubated in order to exchange their VOCs without direct contact with one another. The assay was performed in three replicates. A 4-day old growth plate with *V. longisporum* ELV43 was placed on top of the *P. polymyxa* Sb3-1 plate and sealed to facilitate accumulation of VOCs. For the negative control, a fungal and a bacterial plate were incubated with a non-inoculated PDA plate. Cell lysis was performed using Ribolyser FastPrep-24 (MP Biomedicals, Santa Ana, California, USA) for two times 30 s at 6 m s^−1^ in 90% methanol. The cell free extract was stored at −70°C. The bacterial and fungal metabolite extracts were analyzed with a combined HPLC-hybrid quadrupole-orbitrap mass spectrometer (Q Exactive; Thermo Scientific, Bremen, Germany). A Luna 5u NH2 100A 250 × 4.6 column (Phenomenex, Aschaffenburg, Germany) was used to separate different metabolites from the cell extracts as described by Cernava et al. (2015b). Identification of the soluble compounds was performed with the XCalibur 2.2 and SIEVE 2.2 (Thermo Scientific, Bremen, Germany) and manual comparison of the spectra with corresponding spectra from literature as well as such from mzCloud (HighChem LLC, Bratislava, Slovakia).

### Statistical analysis

The PGP and antifungal effects of the Sb3-1 were statistically analyzed using the IBM SPSS program version 20.0 (IBM Corporation, Armonk, NY, USA). The significance of the differences in the assessed features (germination rate, plants' weights, diameter of the *Verticillium* plugs) between the control vs. each treatment group was calculated using a pairwise *t*-test with independent samples. The decision to make use of the non-parametric Mann–Whitney *U*-test as an alternative to the *t*-test was based an assessment of the distributions of variables (normal vs. non-normal).

## Results

### PGP and antifungal effects of *P. polymyxa* Sb3-1 applied to the seeds of OSR

**Figure 1:**
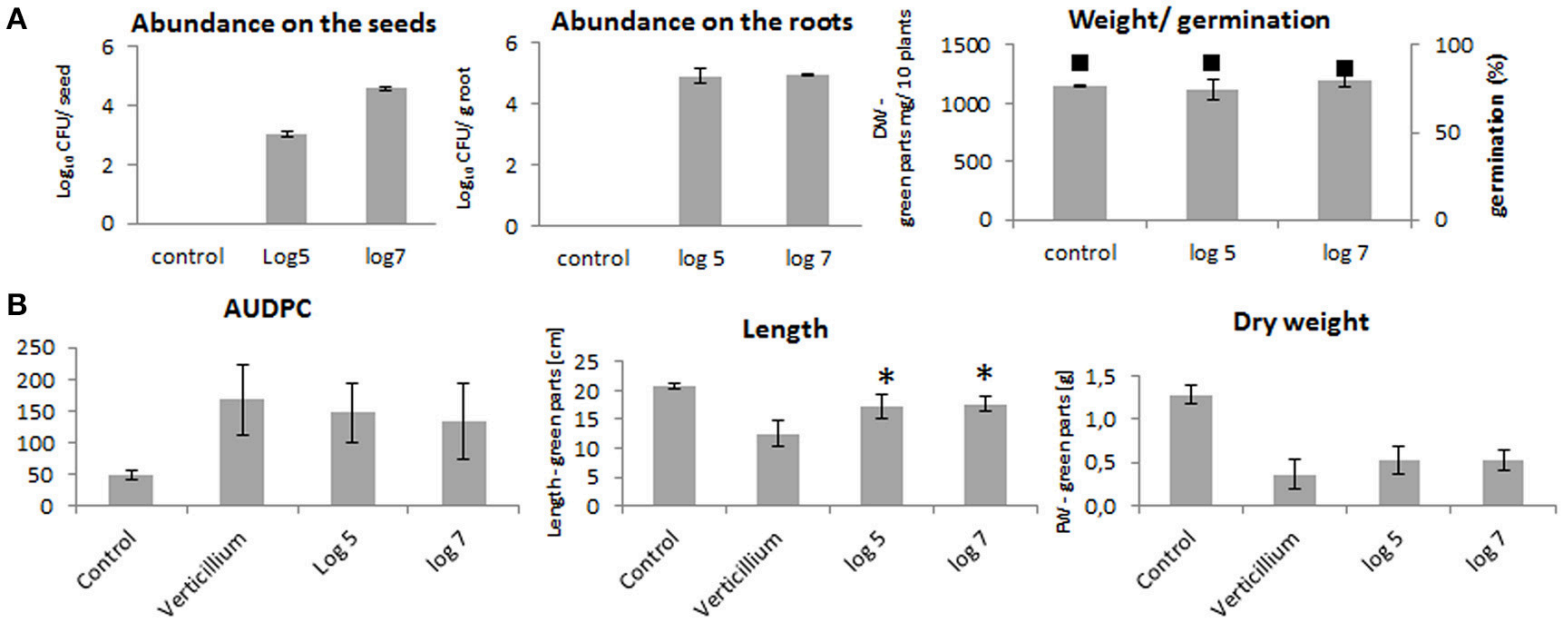
PGP effect on the OSR seedlings and Verticillium wilt disease reduction due to the treatment of the OSR seeds with log_10_ 5 and log_10_ 7 CFU ml^−1^of *P. polymyxa* Sb3-1. **(A)** Abundances of the Sb3-1 on the seeds (left panel) and on the roots (middle panel) as well as fresh weights and germination rates (black squares) of the of the 2-week old seedlings (right panel) treated with Sb3-1 compared to the untreated control (control). **(B)** The Verticillium wilt disease severity (AUDPC; left panel), and lengths and weights (middle and rights panels, respectively) of the 8 weeks old OSR seedlings infected with *V. longisporum*. The labels of the bars refer to the different concentrations of *P. polymyxa* Sb3-1 in the initial inoculant used for seed treatments: log 5, log_10_ 5 CFU ml^−1^ of Sb3-1; log 7, log_10_ 7 CFU ml^−1^ of Sb3-1; control, untreated control; *Verticillium*, untreated seedlings that were infected with *V. longisporum*. Error bars represent confidence interval (*P* = 0.05). The asterisk (^*^) denotes values that were significantly different from the non-treated control group values (*P* < 0.05) defined using pairwise *t*-test or non-parametric Mann–Whitney *U*-test, depending on the distribution of the samples.

The seed treatments with log_10_ 5 and log_10_ 7 CFU ml^−1^ of *P. polymyxa* Sb3-1 rif^R^ resulted in recovery rates of log_10_ 3 ± 0.1 and log_10_ 4.6 ± 0.1 CFU seed^−1^, respectively (Figure 1A). The concentration of Sb3-1 in the roots of the 2-week old seedlings was log_10_ 4.9 ± 0.3 CFU g root^−1^, independent of the initial inoculum concentration. We observed no PGP effect or effect on the germination rate of the Sb3-1 treatment on the 2-week old OSR seedlings (Figure 1A). When the 1-week old seedlings were inoculated with *V. longisporum*, we observed a mild improvement in disease resistance and dry weight as well as a significant increase in the respective lengths of the seedlings treated with *P. polymyxa* Sb3-1 rif^R^ (Figure 1B). The observed increase in seedling length was 36 and 40% for the treatments with log_10_ 5 and log_10_ 7 CFU ml^−1^, respectively, compared to the untreated control infested with *V. longisporum*.

### *P. polymyxa* Sb3-1 and its VOCs inhibit growth of *V. longisporum* ELV43 in the plate assay

**Figure 2:**
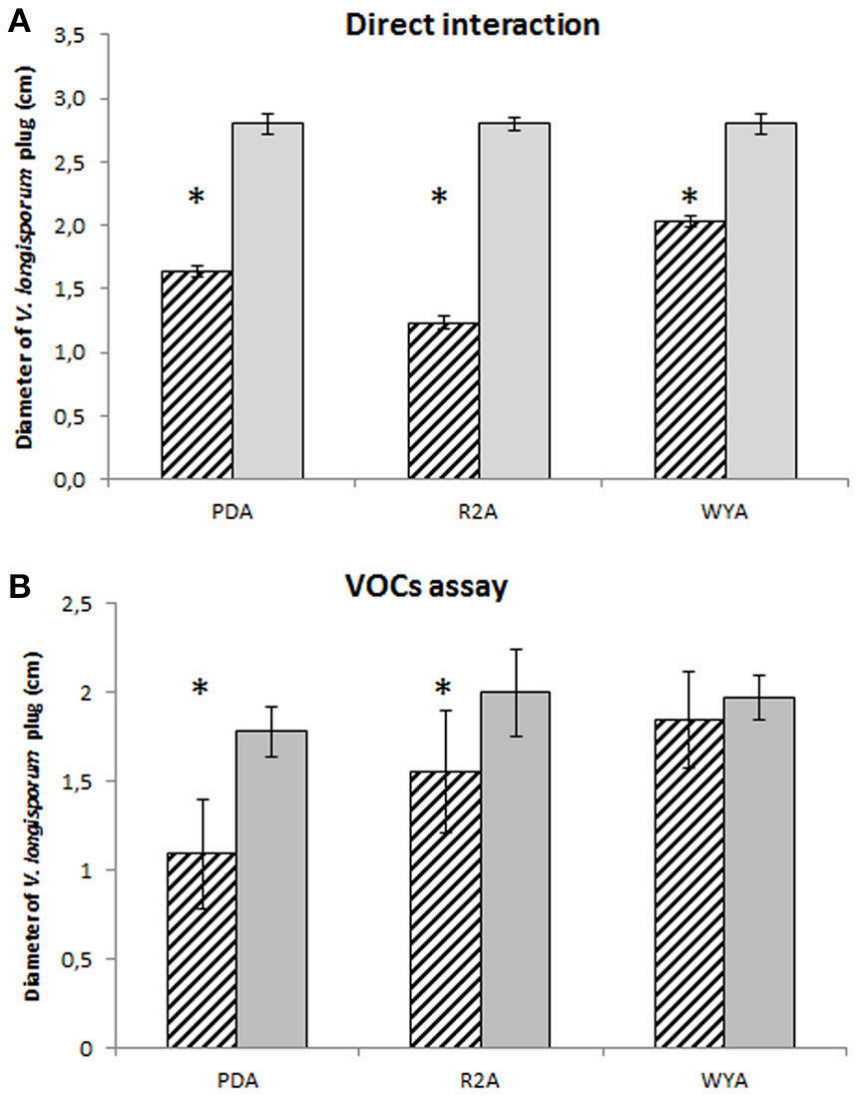
Plate confrontation and VOCs assay of *P. polymyxa* Sb3-1 and *V. longisporum* ELV43 in three different growth media (PDA, R2A, and WYA) for nine days. Dashed columns show growth of *V. longisporum* ELV43 in the presence of *P. polymyxa* Sb3-1 **(A)** or its VOCs **(B)**, while the gray columns illustrate the growth of *V. longisporum* ELV43 without *P. polymyxa* Sb3-1 **(A)** or its VOCs **(B)**. The assay was performed in nine replicates. The asterisk (^*^) denotes values that varied significantly from control group values (*P* < 0.05) defined using pairwise *t*-test or non-parametric Mann–Whitney *U*-test, depending on the distribution of the samples.

In order to understand the mechanism of the antifungal effect of *P. polymyxa* Sb3-1 we designed a series of experiments demonstrating the interaction between *P. polymyxa* Sb3-1 and *V. longisporum* ELV43 *in vitro* on a dual-plate assay and using a specific VOCs assay. After 9 days of co-inoculation the mycelial growth of *V. longisporum* ELV43 was significantly inhibited by *P. polymyxa* Sb3-1 on all media tested (Figure 2A). The incubation of *V. longisporum* ELV43 with the VOCs of Sb3-1 resulted in the significant inhibition of mycelium growth only in combination with PDA and R2A as growth media (Figure 2B). The strongest inhibition of the mycelia growth by *P. polymyxa* VOCs was observed when both microorganisms were grown on PDA plates.

### VOCs involved in the interaction between *P. polymyxa* Sb3-1 and *V. longisporum* ELV43

VOCs produced by *P. polymyxa* Sb3-1 after 3 and 6 days of co-incubation with *V. longisporum* ELV43 volatiles were analyzed using GC-MS headspace SPME, compared to database entries (NIST 2014 database), and verified using covats index (CI; Table 1). Several substances with strong antimicrobial effect, such as three alkyl-substituted pyrazines (Cernava, 2012) were found to be produced after either 3 or 6 days of bacterial growth regardless of the interaction with ELV43 VOCs. We found that higher quantities of known antimicrobial VOCs, such as 2-methyl-1-butanol, hexadecanal (Raza et al., 2015), and isoamyl acetate (Ando et al., 2015), were produced by Sb3-1 after 3 days of incubation than after 6 days of incubation (Table 1). The putatively antimicrobial substances isoamyl acetate and durenol were both downregulated upon contact with *V. longisporum* ELV43 VOCs. We also found two CI-verified substances, 2,3-butanedione and 3-hydroxy-2-butanone 1 within VOCs produced by Sb3-1 that are putatively involved in a bacterial PGP effect (Ryu et al., 2003; Farag et al., 2006). Neither of them was regulated by *V. longisporum* ELV43 VOCs (Table 1).

**Table 1:** GC—MS headspace SPME identification of relevant VOCs produced by *P. polymyxa* Sb3-1 and *V. longisporum* ELV43 grown for 3 or 6 days in presence of each other volatiles.

**RT (min)**	**Predicted compound^a^**	**Regulation^b^**	**Predicted function**
**VOCs PRODUCED BY Sb3-1 AFTER BOTH 3 AND 6 DAYS GROWTH IN PRESENCE OF** ***V. longisporum*** **VOCs**			
8.521	1,3-Dioxolane, 2,2,4,5-tetramethyl-, trans-^*^	↑986%^**^	N.a.
12.238	Pentafluoropropionic acid, hexyl ester^*^	↓47,6%^**^	N.a.
19.248	2-Nonanone^*^	–	Antibacterial (Schulz and Dickschat, 2007)
20.893	2-Decanone^*^	–	N.a.
20.980	2-Decanol^*^	–	N.a.
**VOCs PRODUCED BY Sb3-1 ONLY AFTER 3 DAYS OF CO-INCUBATION WITH** ***V. longisporum*** **VOCs**			
5.25	2,3- Butanedione^*^	–	PGP, antibacterial (Farag et al., 2006; Lee et al., 2012)
5.8	2-Methyl-1-Propanol^*^	–	Antifungal (Stotzky et al., 1976)
6.438	1-Butanol^*^	–	Antibacterial (Létoffé et al., 2014)
7.497	3-Hydroxy-2-butanone^*^	–	PGP, ISR (Ryu et al., 2003)
8.239	2-Methyl-1-butanol^*^	–	Antifungal (Raza et al., 2015)
8.605	3-Methyl-2-pentanone^*^	–	N.a.
12.501	Isoamyl Acetate	↓56%	Antimicrobial (Ando et al., 2015)
13.335	2-Heptanol^*^	–	Antimicrobial (Orhan et al., 2012)
19.621	Decane, 2,6,7 trimethyl^*^	–	N.a.
20.773	**Hexadecanal**	–	Antifungal (Raza et al., 2015)
21.090	Decyl trifluoroacetate^*^	–	N.a.
23.091	Durenol	↓34.1%^**^	Possibly antimicrobial (Al Nomaani et al., 2013)
23.232	2-(2-Methylpropyl)-3- (1-methylethyl) pyrazine	–	Antimicrobial (Aichner et al., 2012)
26.556	Spathulenol	↓44%^**^	Plant metabolite (Pacciaroni et al., 2008)
24.686	2-Dodecanal^*^	–	N.a.
**VOCs PRODUCED BY Sb3-1 ONLY AFTER 6 DAYS GROWTH IN PRESENCE OF** ***V. longisporum*** **VOCs**			
10.25	2-Hydroxy-3-pentanone^*^	–	N.a.
12.25	Pentaflouropropionic acid, hexyl ester^*^	–	N.a.
15.75	2- Isopropylpyrazine	–	Antimicrobial (Aichner et al., 2012)
16.6	3(2H)-Thiononanoe	↑51.5%	N.a.
17.79	1,2-Butanediol, 1-phenyl	–	N.a.
18.74	p-cresol^*^	–	toxic for eukaryotic cells (Andriamihaja et al., 2015)
23.13	2- N-(2-methylpropyl) Benzothiazolamine	↓38.3%	N.a.
23.23	2-(2-Methylpropyl-)-3-(1-methylethyl) pyrazine	–	Antimicrobial (Aichner et al., 2012)
**VOCs PRODUCED BY VL43 AFTER BOTH 3 AND 6 DAYS GROWTH IN PRESENCE OF** ***P. polymyxa*** **Sb3-1 VOCs**			
5.80	Isobutanol^*^	–	Antifungal (Stotzky et al., 1976)
19.90	2-phenylethanol^*^	–	Antimicrobial (Liu and Zhang, 2015)
**VOCs PRODUCED ONLY AFTER 3 DAYS OF CO-INCUBATION WITH** ***P. polymyxa*** **Sb3-1 VOCs**			
7.65	Acetoin^*^	–	PGP, ISR (Ryu et al., 2003)
13.34	2-(4-Cyclohexyl-butanoylamino) -3-chloro-1,4-naphthoquinone	↑100%^**^	Putatively antifungal (Sasaki et al., 2002)
17.792	Bicyclo (2.2.1)-hepta-2,5-dien-7-ol; or 7-Hydroxynorbornadiene	↑100%^**^	N.a.
**VOCs PRODUCED ONLY AFTER 6 DAYS GROWTH IN PRESENCE OF** ***P. polymyxa*** **Sb3-1 VOCs**			
6.44	1-Butanol^*^	↑53.4%	Antimicrobial (Létoffé et al., 2014)
8.10	Isoamyl alcohol^*^	–	Antimicrobial (Ando et al., 2015)
9.23	1,2,4- Benzenetricarboxylic acid, 1,2 dimethyl ester	↑100%^**^	N.a.
17.793	Nα, Nω-Dicarbobenzoxy-L-arginine	↑100%^**^	N.a.
18.08	2-Nonanone^*^	–	Antifungal (Raza et al., 2015)
19.98	3-Phenyl-5-(benzylthio)isoxazole	↑17.9%	N.a.
25.84	5,6-Decadien-3-yne,5,7-diethyl	↑20%	N.a.

a The selection of substances included in the Table was performed as followed: (1) only substances with match index with the NIST MS Search 2.2 included in the Software-Package of the NIST 2014 database over 500 were considered; (2) only the substances that were either verified using covats index [ < 15; labeled with asterisks (

* *)] or showed an up- or down-regulation due to the presence of the VOCs of the other microorganism were included in the Table. The predicted functions of the substances are highlighted in color as follows: the substances with predicted antimicrobial activity are shown in light red, substances with putatively plant growth regulating functions are light green and unknown substances are highlighted in gray*.

b *Regulation in presence of microorganism's VOCs (%). Arrow down (↓) indicates the significant down-regulation of the substance, while the arrow up (↑) represents the significant up-regulation of a substance. A minus sign (“–”) denotes that no significant up- or downregulation of the substance in the presence of the VOCs was detected. The VOCs that were identified by Raza et al. (2015) as produced by P. polymyxa WR-2 are highlighted in bold*.

The double asterisk(

** *)denotes values that were significantly different from the non-treated control group values (P < 0.05) defined using pairwise t-test or non-parametric Mann–Whitney U-test, depending on the distribution of the samples*.

GC-MS headspace SPME analysis revealed that *V. longisporum* ELV43 produced several substances with known antimicrobial properties such as isobutanol (Stotzky et al., 1976) and 2-phenylethanol (Liu and Zhang, 2015). These substances were detected after 3 and 6 days of *V. longisporum* growth. Acetoin, a substance with a known PGP effect that also induces systemic resistance and regulates auxin homeostasis in *A. thaliana* (Ryu et al., 2003), was detected in the volatile phase after 3 days of *V. longisporum* growth. Seven VOCs were upregulated in ELV43 during co-incubation with *P. polymyxa* Sb3-1 volatiles (Table 1). Two of them, a naphthoquinone-derivate and 1-Butanol, both exhibiting putative antimicrobial effect (Sasaki et al., 2002; Létoffé et al., 2014) were upregulated due to contact with Sb3-1 VOCs (100 and 53.4%, respectively).

### Regulation of soluble metabolites produced by *P. polymyxa* Sb3-1 and *V. longisporum* Elv43 as a reaction to each other VOCs

The production of soluble metabolites by both microorganisms, *P. polymyxa* Sb3-1 and *V. longisporum* ELV43 in response to one another's VOCs was studied by means of high-resolution LC-MS analyses. More than 100 substances were detected in the *P. polymyxa* Sb3-1 samples (data not shown). Of the substances that we were able to be identified with a high degree of certainty (based on NIST 2014 database searches), several were differentially regulated due to the interaction with ELV43 VOCs. Two amino acids, valine and glutamic acid, were upregulated in the 3-day old culture of Sb3-1 due to its exposure to *V. longisporum* EVL43 volatiles, while one amino acid derivative, aminocaproic acid, was downregulated (Table 2). Six days after inoculation, several substances involved in the cellular metabolism, such as amino- and nucleic acids were upregulated. Only two substances, citric acid and an unidentified substance, were downregulated because of Sb3-1 exposure to ELV43 VOCs. Additionally, surfactin was detected in every sample of *P. polymyxa* Sb3-1 regardless of the EVL43 VOCs exposure. Surfactin is known for its biosurfactant and antibacterial properties (Pratap et al., 2013) and for its ability to activate the formation of biofilm (Timmusk et al., 2015). Spermidine, a stress protection agent (Kusano et al., 2008), was found after 6 days of Sb3-1 growth, while antimicrobial fusaricidin (Kajimura and Kaneda, 1996) was found after both 3 and 6 days of bacterial growth. Both components were not regulated by EVL43 VOCs. Interestingly, other well-known antimicrobial *Paenibacillu*s metabolite polymyxin was not detected under the conditions tested in any of the samples.

**Table 2:** Effect of *V. longisporum* ELV43 VOCs on *P. polymyxa* Sb3-1 metabolism detected by LC-MS.

**3 days of co-incubation^a^**	**Putative function**	**Regulation^b^**
Valine^*^	Protein component	↑
Glutamic acid^*^	Protein component	↑
Aminocaproic acid^*^	Lysine derivate/protein degradation	↓
**6 days of incubation**^a^	**Putative function**	**Regulation**^b^
Adenine^*^	DNA component	↑
Unknown structure (C_35_H_47_NO_8_)^*^	n.a.	↓
Guanosine^*^	Lipoic acid metabolism	↑
Adenosine^*^	DNA component	↑
L-Threonyl-L-leucin^*^	Glycine, serine and threonine metabolism	↑
DL-Methionine^*^	Protein component	↑
DL-Glutamic acid^*^	Protein component	↑
Boc-L-glutamine (N~2~-(tert-Butoxycarbonyl)glutamin)	Glutamate derivate/protein degradation	↑
Citric acid^*^	General metabolism	↓
Bengamide derivate (2R,3R,4S,5R,6E)-3,4,5-Trihydroxy-2-methoxy-8-methyl-N-[(3S)-1-methyl-2-oxo-3-azepanyl]-6-decenamide)	Potentially cytotoxic, anticancer activity (Thale et al., 2001)	↑

a *Only verified substances that were up- or down-regulated by V. longisporum ELV43 VOCs are shown*.

b Arrow up (↑) symbolizes that the corresponding substance is upregulated, while arrow down (↓) symbolizes a downregulation by V. longisporum ELV43 VOCs. N.a. (not available) means that a putative function could not be found. Asterisk (

* *) symbolized that the up- or downregulation was statistically significant (p < 0.05)*.

Analysis of *V. longisporum* ELV43 metabolites showed that 3 days of incubation with *P. polymyxa* Sb3-1 VOCs lead to up-regulation of L-Isoleucyl-L-glutamic acid, an incomplete breakdown product of protein digestion. On the other hand, the concentration of several metabolites involved in cellular metabolism (e.g., several amino acids and intermediates in the amino acid synthesis), of a substance involved in the cell wall formation (DL-2,6-Diaminopimelic acid) as well as of pantothenic acid, decreased (Table 3). Interestingly, after 6 days of co-incubation with *P. polymyxa* Sb3-1 volatiles, the upregulation of detected substances prevailed over downregulation. We detected upregulation of arabitol, a carbohydrate involved in cell stress response, and several breakdown products of protein and RNA biosynthesis. On the other hand, N-Acetyl-L-Carnosine (known as an antioxidant) and cystathionine were downregulated following exposure to Sb3-1 VOCs.

**Table 3:** Effect of *P. polymyxa Sb3-1* VOCs on *V. longisporum ELV 43* metabolism detected by LC-MS.

**3 days of co-incubation^a^**	**Putative function**	**Regulation^b^**
DL-Histidine^*^	Protein component	↓
DL-Proline^*^	Protein component	↓
DL-Glutamic acid^*^	Citric acid cycle/Protein component	↓
L-Isoleucyl-L-glutamic acid^*^	Protein breakdown product	↑
Cystathionine^*^	Cystein synthesis intermediate	↓
DL-Glutamine^*^	Protein component	↓
L-Saccharopine^*^	Lysine synthesis intermediate	↓
DL-2,6-Diaminopimelic acid	Cell wall component	↓
Pantothenic acid^*^	Vitamine	↓
**6 days co-incubation**^a^	**Putative function**	**Regulation**
Arabitol^*^	Sugar/response to cell stress	↑
L-Isoleucyl-L-glutamic acid	Protein breakdown product	↑
Methylglutaric acid^*^	n.a.	↑
Uridine^*^	RNA component	↑
N-Acetyl-L-Carnosine	Antioxidant	↓
Cystathionine^*^	Intermediate in the synthesis of cysteine	↓

a *Only verified substances that were up- or down-regulated by V. longisporum ELV43 VOCs are shown*.

b Arrow up (↑) symbolizes that the corresponding substance is upregulated, while arrow down (↓) symbolizes a down regulation. n.a. (not available) means that a putative function could not be found. Asterisk (

* *) symbolized that the up- or downregulation was statistically significant (p < 0.05)*.

## Discussion

The current study underpins the importance of volatiles as mediators of microbial interactions. Moreover, the results suggest that microbial VOCs from beneficial bacteria can induce substantial changes in target pathogens. *P. polymyxa* Sb3-1 was able to reduce the growth of *V. longisporum* ELV43 *in vitro* directly, and via its volatiles. The antifungal effect of Sb3-1 was confirmed *in planta* by a significant improvement in the lengths of the oilseed rape seedlings treated with Sb3-1 compared to the untreated control. The observed plant growth promotion effect was only detected when the seedlings were infested with *V. longisporum*. This suggests that the antagonistic properties of the *Paenibacillus* strain or its ability to induce resistance in the host plant were responsible for the beneficial effect on the plant growth. The production of a biosurfactant and activator of biofilm formation surfactin (Pratap et al., 2013; Timmusk et al., 2015) as well as the stress protection agent spermidine (Kusano et al., 2008) and antimicrobial fusaricidin by Sb3-1 may enhance the PGP effect under biotic stress conditions. The production of 2,3-butanedione and 3-hydroxy-2-butanone by Sb3-1 may also explain PGP properties of Sb3-1 observed in our greenhouse experiments and in previous *in planta* tests under sterile soil conditions (Rybakova et al., 2016b).

**Figure 3:**
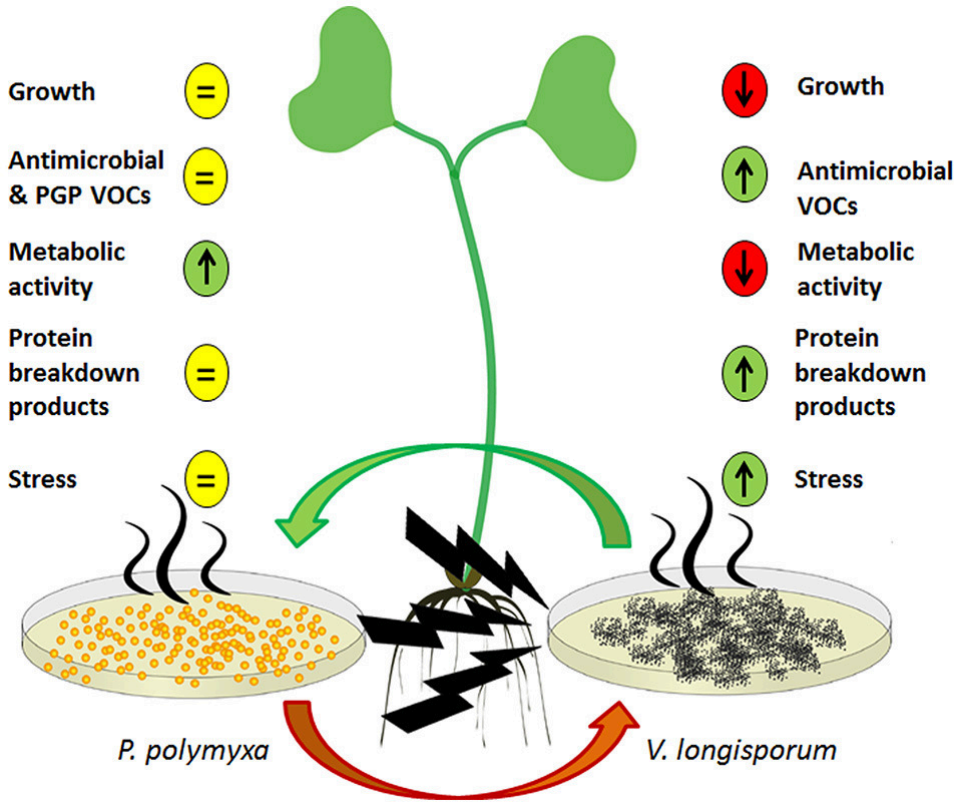
A model of a dialog between *P. polymyxa* Sb3-1 (left) and *V. longisporum* (right) via their VOCs and their influence on the host plant (middle). The predicted up- and downregulated processes are shown as up- and down arrows, respectively. The non-regulated processes are depicted as an equal symbol.

The majority of our results revealed contrasting effects with regards to the ways in which the two microorganisms reacted to one another's VOCs as illustrated by the model in Figure 3. While *P. polymyxa* Sb3-1 was not visually impaired in its growth due to ELV43 volatiles, the growth rate of *V. longisporum* ELV43 was significantly reduced. Both of the microorganisms constantly produced potentially antimicrobial volatiles; however the production of some antimicrobial VOCs by Sb3-1 was reduced upon contact with ELV43 VOCs, while ELV43 demonstrated upregulation of antimicrobial VOCs due to the interaction with Sb3-1. This may indicate that *P. polymyxa* does not “regard” *V. longisporum* as an enemy and slows its defense system down, while the fungus switches on some type of additional defense mechanism to counterattack the antimicrobial VOCs of Sb3-1. A similar effect was observed when we studied soluble metabolites of both interacting microorganisms. While *P. polymyxa* Sb3-1 demonstrated upregulation of some essential amino acids and DNA components, the opposite was found for *V. longisporum* ELV43. This finding may reflect a change in the metabolic rate of both microorganisms as a reaction to one another's VOCs. There are indications that the metabolism of *V. longisporum* ELV43 slows down due to the contact with damaging Sb3-1 VOCs as the cells undergo an apoptotic process. This was also confirmed by the observed accumulation of protein breakdown products and a specific carbohydrate indicating cellular stress (arabitol) by ELV43 exposed to Sb3-1 VOCs. In contrast, the metabolic activity of Sb3-1 seems to be activated by the ELV43 VOCs as is suggested by accumulation of protein and DNA components by ELV43.

Interestingly, out of 40 identified VOCs produced by *P. poly*myxa Sb3-1 only three (2-nonanone, 2-methyl-1-butanol, and hexadecanal) were identical with those identified by Raza et al. (2015) in *P. polymyxa* WR-2 VOCs. This strain was found to be active against *Fusarium oxysporum* f. sp. niveum *in vitro*. The variations in the composition of volatiles might be primarily due to differences in the growth media compositions, however the structure of *Paenibacillus*' “volatilomes” can also vary to a certain degree as highlighted by Rybakova et al. (2016a). In addition, it indicates a very specific pathogen-antagonist interaction. We first detected one putative PGP substance acetoin (3-hydroxy-2-butanone), which was also found to trigger induced systemic resistance in *Arabidopsis* (Rudrappa et al., 2010) produced by the plant pathogen *V. longisporum* ELV43. Although, this finding needs to be further investigated, it may also explain the plant-beneficial effect described for non-pathogenic *Verticillium* strains (França et al., 2013). Furthermore, it also supports the theory that microbial diversity is crucial for combating “microbiome diseases” such as Verticillium wilt because *Verticillium* species occur frequently in healthy plants and contribute to the functioning of the holo-biont (Berg et al., 2017).

In conclusion, our data indicates that *P. polymyxa* Sb3-1 and *V. longisporum* ELV43 are in a constant dialog with one another via their VOCs. This specific dialog results in the inhibition of cellular metabolism in *V. longisporum* ELV43 leading to the growth reduction of the fungus. This antagonistic effect in addition to the production of PGP volatiles leads to an overall positive effect on the growth of the infested host plant. This study contributes to the better overall understanding of the interactions between the potential BCA *P. polymyxa* Sb3-1 and the plant pathogen *V. longisporum*. It supports the effort to develop this beneficial bacterium into a viable BCA against Verticillium wilt, and offers a promising option in the progress toward realizing sustainable agriculture in the context of global warming.

## Author contributions

GB and DR designed the study. UR carried out the *in vitro*, LC-MS and GC-MS assays and analyzed the GC-MS data. MS and DR carried out plant growth promoting and biocontrol assays. AS did the analytic for LC-MS assays. TC and AS consulted on the GC-MS assays and TC contributed to the VOCs-related parts of the final manuscript. DR wrote the final version of the manuscript with input from GB, TC, and UR. All authors read and approved the final manuscript.

### Conflict of interest statement

The authors declare that the research was conducted in the absence of any commercial or financial relationships that could be construed as a potential conflict of interest.
