# Supplementary material for: Aerial Warfare: A Volatile Dialogue between the Plant Pathogen Verticillium longisporum and Its Antagonist Paenibacillus polymyxa
Source: Front Plant Sci. 2017 Jul 27;8:1294. doi: 10.3389/fpls.2017.01294 (PMC5529406; doi:10.3389/fpls.2017.01294)
Supplement: Supplementary file 5 [file DataSheet5.DOCX]

Supplementary Material

**Aerial Warfare: a volatile dialogue between the plant pathogen *Verticillium longisporum* and its antagonist *Paenibacillus polymyxa***

**Daria Rybakova^1*^, Ute Rack-Wetzlinger^1^, Tomislav Cernava^2^, Angelika Schaefer^1^, Maria Schmuck^1^ and Gabriele Berg^1^**

*** Correspondence:** daria.rybakova@tugraz.at

# Supplementary Data

The raw data of GCMS analyses publication is provided as .zip files. Filenames include the analyzed microorganisms and the duration of the incubation prior to VOC sampling as follows:

GCMS data 3 day Verticillium incubations.zip

GCMS data 3 day Paenibacillus incubations.zip

GCMS data 6 day Verticillium incubations.zip

GCMS data 6 day Paenibacillus incubations.zip

# Supplementary Figures

#
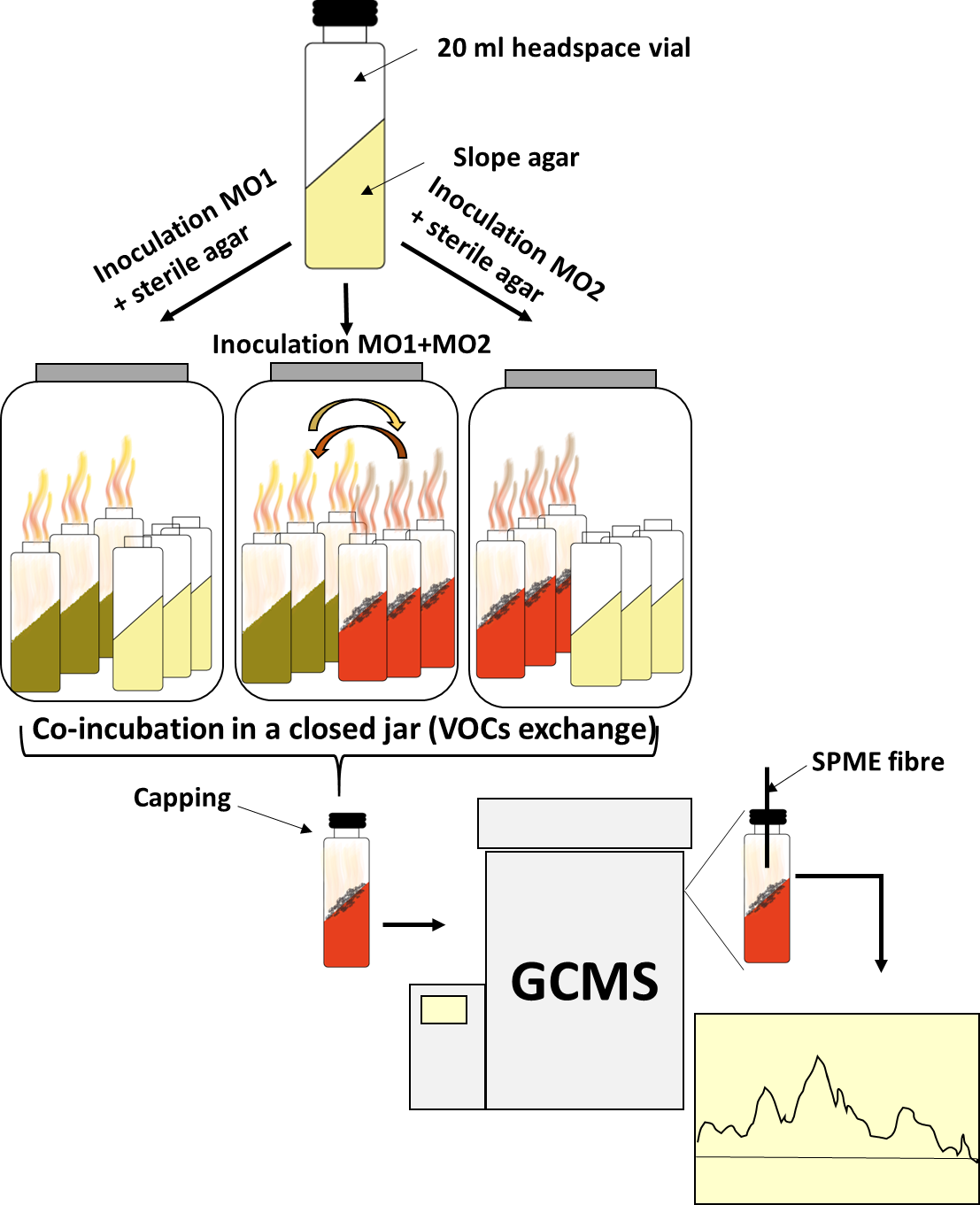


**Supplementary Figure 1.** The layout of the GC-MS headspace SPME experiment. Both microorganisms were grown separately on the slope agar. The vials containing each microorganisms were co-incubated with each other or with an uninoculated vial (negative control) without leads in a closed jar, allowing the volatile compounds to interchange. After the incubation period the jars were opened and the vials closed. The produced VOCs in each vial were analysed using GCMS.
