# Supplementary material for: Aerial Warfare: A Volatile Dialogue between the Plant Pathogen Verticillium longisporum and Its Antagonist Paenibacillus polymyxa
Source: Front Plant Sci. 2017 Jul 27;8:1294. doi: 10.3389/fpls.2017.01294 (PMC5529406; doi:10.3389/fpls.2017.01294)
Supplement: Supplementary file 6 [file Image1.PDF]

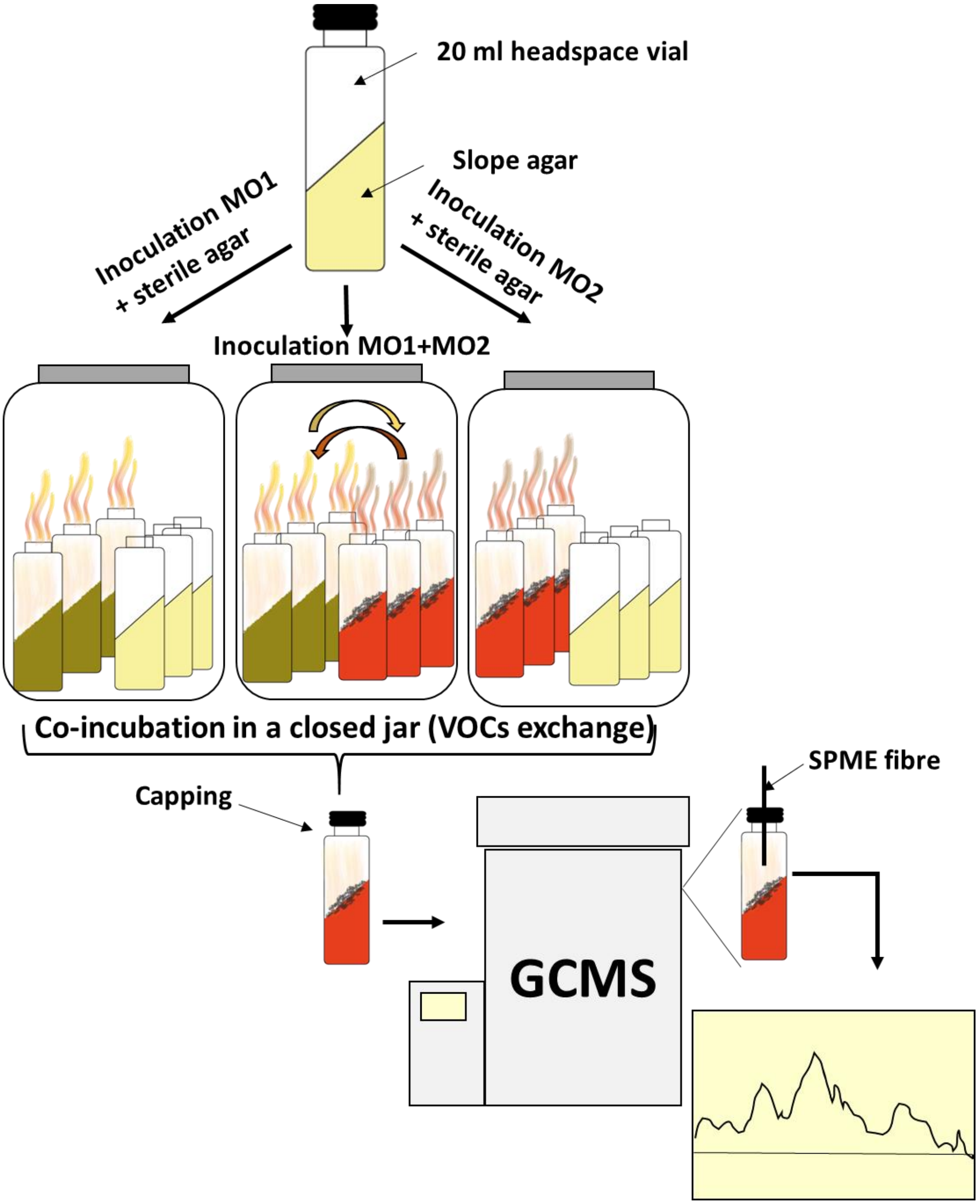

Supplementary Figure 1. The layout of the GC-MS headspace SPME experiment. Both microorganisms were grown separately on the slope agar. The vials containing each microorganisms were co-incubated with each other or with an uninoculated vial (negative control) without leads in a closed jar, allowing the volatile compounds to interchange. After the incubation period the jars were opened and the vials closed. The produced VOCs in each vial were analysed using GCMS.
